# Development and validation of the parents’ cognitive perception inventory of disaster effects on children’s well-being (PCP-DCWB)

**DOI:** 10.1186/s40359-022-00918-1

**Published:** 2022-09-03

**Authors:** Najibeh Atazadeh, Hassan Mahmoodi, Parvin Sarbakhsh, Abdolreza Shaghaghi

**Affiliations:** 1 Present Address: Department of Health Education and Promotion, Faculty of Health, Tabriz University of Medical Sciences, Golgasht Ave., Tabriz, Iran; 2 Social Determinants of Health Research Center, Research Institute for Health Development, Kurdistan University of Medical Sciences, Sanandaj, Iran; 3 Department of Epidemiology and Biostatistics, Faculty of Health, Tabriz University of Medical Sciences, Golgasht Ave., Tabriz, Iran; 4 Department of Health Education and Promotion, Faculty of Health, Tabriz University of Medical Sciences, Golgasht Ave., Tabriz, Iran

**Keywords:** Trauma effects, Children’s well-being, Measure development, Parents’ cognition

## Abstract

**Background:**

Parents’ cognition about the type and nature of consequences a disaster may pose on the children’s psychosocial health, could be a major protective factor against the long-term overwhelming complications. Given the lack of a reliable instrument to measure parents’ cognition about disasters’ effects on children’s well-being, this study was conducted to develop and validate the parents’ cognitive perception inventory of disaster effects on children’s well-being (PCP-DCWB).

**Methods:**

In this cross-sectional study 300 parents of the survived primary school aged children from the Iran’s northwest earthquake on August 2012 were recruited in the city of Varzegan. Exploratory factor analysis (EFA) was applied to identify the subcomponents and Cronbach’s alpha and Guttmann Split-half coefficients were calculated to assess the internal consistency reliability of the scale.

**Results:**

Structural indicators of the Kaiser–Meyer–Olkin measure (0.69) and Bartlett’s test of Sphericity (*P* < 0.001, *df* = 153, X^2^ = 618.35) verified interpretability of the EFA output. Applying principal component analysis and direct oblimin rotation in the EFA four latent factors were identified (i.e., perception about child overall mental health, coping with trauma’s long-term effects, children or parents’ continuing memory of past disaster and perception about behavioral and educational problems) which explained 49.32% of the total variance. The estimated Cronbach’s alpha and split-half reliability coefficients (0.71 and 0.52 respectively) supported good internal consistency of the instrument.

**Conclusion:**

The study findings revealed sound psychometric attributes of the PCP-DCWB to be applied in assessment of parents’ cognition about psychological impacts of a traumatic event on the survived children. The instrument application can shed light on level of pre-disaster preparations in local, national and international scales and help effectiveness assessment of interventions that target maintenance of psycho-social well-being among disaster-affected survivors over time.

## Introduction

Natural disasters may pose devastating impacts on mental health and social well-being of survivors by threatening personal security, defense mechanisms repression and disrupting the family’s structure [1]. Children are more prone to the psychological consequences of disasters however; the severity of symptoms depends on a number of distinct factors such as the level of exposure to accident, destruction or loss of home, personal injury, decease of family members and friends, witnessing a death scene, feeling trapped and disabled, separation from parents and the level of parental support [2, 3].

Parental care conceivably plays an influential role in children's health, since it regulates most interactions of a child’s environment and helps children in better adapting to the circumstances of socio-physical environment [4]. Disasters generally influence family members’ functioning, but children may be at added risk of secondary psychic trauma by virtue of their parents or siblings’ emotional reactions to the adverse event. Findings of research on earthquake survivors have revealed that when parents become irritable and disturbed as a result of the post-traumatic stress disorder (PTSD), odds of occurrence and severity of a relentless psychological reactions to the circumstantial stresses could also escalate in their children [5]. Therefore, children’s post-trauma well-being was suggested to be highly dependent on their parents’ reactions to disasters, coping and resilience capacities and being knowledgeable about the potential impact of a disaster on children’s psychological well-being [6].

Widespread increase in frequency and severity of disasters around the world from one hand; and emotional and psychosocial impacts of natural disasters on children (e.g. post-traumatic stress disorder (PTSD), depression, anxiety, emotional and behavioral difficulties and other mental health disturbances) [7–13] that might be expansive in scope and extend into their later life at the other hand, heralds the need for paying further attention to the post-trauma well-being of children [14, 15]. Children of families with improved resilience capacity for problem solving generally have a better overall competency in responding even to severe crises [16].

Social norms could have influence on mental health issues and coping mechanisms in communities when people are confronting with stressful life events. Scale of disintegration and disruption of social norms and support structures following a disastrous event can be different according to the inherent nature of the occurred disaster and degree of communities’ pre-disaster preparation. There is a plethora of research evidence that verifies positive impact of trauma-informed parents on the child survivors’ well-being [17–23]. Knowledgeable parents could better support development of their offspring’s’ resilience skills in coping with disasters, therefore they could play a central role in maintaining the integrity of an expeditious rescue operation. Studies shown that parents’ perception and awareness about signs of psychological disorders in children following a disastrous event is an important precedent for early detection and treatment seeking for the condition [24].

Current global pattern of disasters’ frequency and severity implies the need for a reliable data collection tool to measure parents’ perceptions about the type and nature of consequences a disaster may pose on the children’s psychological serenity. This can be a major step in planning interventions that target prevention of disruption in the child survivors’ overall well-being.

Iran with a population of about 83 million is one of the most often disaster-hit countries of the Middle East and North Africa region [25]. The country is seismically active with three active fault lines that run through 77 percent of the country’s urban areas [26]. According to the registered data a major earthquake is taken place every 2–3 years in Iran in addition to other major natural disasters that include floods, droughts, desertification, deforestation and storms [26, 27]. These disastrous events have potential to engender serious and long lasting impacts on the survivors’ well-being especially children. Recognition of measures or factors that could alleviate disasters’ burden on children therefore, has surpassing implication for an evidence informed disaster preparedness programing.

Different tools and scales specifically designed to measure one aspect of post-trauma psychological problems (e.g. prolonged fear, stress, anxiety) in children, but to the best of current knowledge a validated questionnaire to measure self-perceived cognition of parents regarding the consequences of disasters on children’s well-being do not exist [28, 29]. Given the lack of a reliable instrument to measure parents’ cognition about the consequences of disasters on children’s psychological well-being this study was conducted to develop and assess reliability and validity of the Parents’ Cognitive Perception Inventory of Disaster Effects on Children’s Well-being (PCP-DCWB).

## Materials and methods

### Study location, participants and procedure

The recommended procedures explained by Robert F. DeVellis [30] for development of new measurement tools were utilized to construct the PCP-DCWB. The study was performed in the city of Varzegan, north of East Azarbaijan province and in the stricken zone of the Iran’s northwest earthquake on August 2012 [31]. Self-completion data collection method was employed to obtain the study data from 300 parents of survived school children (n = 150) based upon their mutual consensus about the provided responses to the scale’s items from October 2015 to April 2016.

### Items’ generation

A multi-stage approach was administered to design and assess psychometric properties of the PCP-DCWB. At the first stage an extensive literature review regarding children’s psychological problems and patterns of parental care, the role of parents in caring for their children and parental assessment of children’s reactions was performed to develop a preliminary topic list of the probable items in a culturally acceptable manner [3, 32–34]. Correct and careful judgment of parents about psychological well-being of their disaster-impacted children is dependent on having a sound mental health state per se. Therefore, items related to mental well-being of a respondent parent were also decided to be included in the scale. The outputs helped elaboration of a topic list and allocation of items relevant to the extracted topics based upon the best synthesis of identified factors in the existing literature.

A multidisciplinary expert panel of ten psychologists, clinical psychiatrists and health education and promotion specialists who had been recruited through purposive sampling method evaluated the face and content validity of the generated items in the first draft of the questionnaire. The panelists were chosen based on their experiences and were insightful about disasters’ effects on well-being of survivors. The importance and need for a simple and concise tool in exploration of parents’ cognitive perceptions about disasters’ effects on well-being of child survivors was briefly explained to the recruited informants.

### Items’ analysis and reduction

#### Content validity

The panelists were requested to independently rate the relevance, clarity, importance and simplicity of each item on the PCP-DCWB by using a direct continuous 4-point Likert scale. They were also asked to give their feedback regarding the individual items or the whole instrument. Items were analyzed based on a formal consensus process after iterations of independent ratings by the respondents. To evaluate the experts’ agreement on the content validity, item-level content validity index (I-CVI) was calculated considering their judgments about the importance, relevance and clarity of the items. I-CVI represents the proportion of experts that arrived at an acceptable test rating (3 or 4 to indicate an item is quite or highly important, relevant and clear) by the total number of assessments. Items with an I-CVI of 0.78 or higher were considered to have good content validity in this study [35]. Thus; 18 items incorporated after refining and eliminating of redundant items from the provisionary list of 21 items. The final instrument consisted of 7 items to measure the domain of self-perception of disasters’ psychological impacts (items 1–7 e.g. *“I am hopeful about the future”*, *“Despite being exposed to the quake, I feel that I have an adequate control of my living conditions”*, *“I can easily tell my feelings as well as circumstances, which I had after the quake, to other people”* etc.) and 11 items (items 8–18 e.g. *“I feel that my child has become more fearful after the quake”*, *“I feel that my child is always anxious”*, *“My child is become more dependent on us to do daily tasks such as bathing or cleaning”* etc.) to examine perceptions about disasters’ burden on well-being of children.

#### Items’ scoring

The response choices for each of the 18 items were considered as; "very often", "often", “sometimes”, “infrequently” and "never". The scoring of 5, 4, 3, 2 and 1 attributed to each of these responses in questions 1–5 and 7 so that the higher value indicated a better self-perceived psychological well-being of the interviewees. The reverse scoring system was applied to the responses in the questions 6 and 8–18 so that the higher value [5] indicated a lower likelihood of reporting of a post disaster psychological problem in the respondent or her/his child and the lower value [1] represented a higher likelihood of having a problem in well-being. Therefore; the total score ranged from 18 to 90 so that a higher overall scale score represents the interviewee’s better level of perception about induced psychological problems in their disaster-affected child.

#### Translation process

The original draft of the instrument developed in English and translated into Persian independently by two professional translators and then back translated to English to ensure accuracy of the translation and cross-language comparability of meaning. All necessary amendments or modifications were carried out at this stage.

#### Pilot test

The Persian version of the developed instrument was pilot tested on a convenience sample of 20 parents of children who survived the Iran’s northwest earthquake on August 2012 [31]. The questionnaire was disseminated to the respondents through their school-aged children who had been recruited from the local schools. The approached parents were asked in a cover letter to complete the scale and also give their comments on items that were confusing, difficult to understand or respond. Their feedbacks were considered carefully to improve clarity and relevance of items and necessary modifications were made subsequently.

#### Psychometric assessment

Psychometric assessment of the PCP-DCWB was performed by application of stratified sampling technique to recruit surviving parents of 150 enrolled primary school aged children (grades 3–5) in the two primary schools within the city of Varzegan that is located in the 2012 Iran’s northwest earthquake-strike zone. The sample size was ascertained based on the recommended sample size for a multivariate analysis in instrument validation studies [36].

Two copies of the PCP-DCWB attached to an information sheet to describe the study purpose and informed consent forms were sent to the parents and they were asked to return the completed questionnaires with informed consent forms in a pre-stamped sealed envelope if they are willing to participate in the study. A reminder call was attempted to all non-respondents 2 weeks after the initial mail out. Several rounds of follow up were also carried out for those who did not return the questionnaire within 4–5 days after the initial reminding calls. Collection of all distributed questionnaires took about 43 days. Those parents who themselves or their child had a severe disabling physical or mental disorder and single parent families were excluded from the study to alleviate probability of confounding bias.

### Scale validation

Exploratory factor analysis (EFA) using principal component analysis (PCA) and oblimin rotation with Kaiser normalisation method was carried out to determine presence of underlying dimensional structure in the parents’ cognitive perception about disaster effects on children’s well-being. The Kaiser–Meyer–Olkin (KMO) measure and Bartlett test of Sphericity were also used to asses sampling adequacy and factorability of the study data.

The internal consistency reliability of the developed scale was appraised by employing the Cronbach’s alpha coefficient and Guttmann Split-half test results*.* All participants were assured about confidentiality of their identity and the collected study data before signing the informed consent form.

## Results

Mean (SD) age of the study participants was 36.42 (4.68), 5.3% were illiterate and almost 60.8% of them had below the secondary school educational level. Detailed socio-demographic characteristics of the studied sample were shown in Table 1.

**Table 1:** General characteristics of the recruited students/parents in the validation study of parents’ cognitive perception inventory of disaster effects on children’s well-being (PCP-DCWB)

Studied groups	Number	Mean age	Standard deviation	Age range
*Students*				
Boys	75	10.36	1.07	9–12
Girls	75	10.58	1.06	
Total	150	10.47	1.07	
*Parents*				
Fathers	150	38.68	4.23	29–48
Mothers	150	34.16	3.98	24–44
Total	300	36.42	4.68	24–48

Parents education	Mothers			Fathers		
	Number	%	Cumulative %	Number	%	Cumulative %
Illiterate	6	4.0	4.0	2	1.3	1.3
Writing and reading skill	2	1.3	5.3	5	3.3	4.6
Primary	45	30.2	35.5	31	20.7	25.3
Secondary	36	24.2	59.7	35	23.3	48.6
High School	48	32.2	91.9	53	35.4	84.0
Associate degree	7	4.7	96.6	8	5.3	89.3
Bachelor degree	5	3.4	100.0	13	8.7	98.0
Post graduate degree	0	0	100.0	3	2.0	100.0

The estimated item level content validity index (I-CVI) which was calculated based on the experts’ panel feedback was in the range of 0.7 to 0.93. Those items with I-CVI values in the range of 0.7–0.78 were reassessed and revised for clarity according to suggestions made by the key informants and therefore retained to be included in the scale. The referred panelists confirmed all of the 18 items therefore; they were deemed appropriate for inclusion in the rest of the psychometric procedure. Based on the parents provided responses intercorrelations of the item scores were tabulated in Table 2.

**Table 2:** Inter-correlation of the items in the validation study of parents’ cognitive perception inventory of disaster effects on children’s well-being (PCP-DCWB)

Items	1	2	3	4	5	6	7	8	9	10	11	12	13	14	15	16	17	18
1	1																	
2	.349^**^	1																
3	.362^**^	.359^**^	1															
4	.309^**^	.455^**^	.434^**^	1														
5	.240^**^	.452^**^	.254^**^	.325^**^	1													
6	− .056	− .175^**^	.094	.002	− .277^**^	1												
7	.232^**^	.078	.202^**^	.108	.009	.186^**^	1											
8	− .124^*^	− .014	− .047	.023	− .199^**^	.148^*^	.000	1										
9	− .107	− .034	− .104	.000	− .100	.163^**^	.077	.417^**^	1									
10	− .148^*^	− .123^*^	− .133^*^	− .111	− .283^**^	.262^**^	.015	.430^**^	.481^**^	1								
11	− .037	− .055	− .145^*^	− .115^*^	− .086	.092	− .016	.090	.254^**^	.180^**^	1							
12	.120^*^	.126^*^	.092	.306^**^	.004	.050	.049	.047	.104	.083	.020	1						
13	.096	.054	.069	.174^**^	− .110	.062	.042	.037	.080	.071	.050	.154^**^	1					
14	− .109	− .039	− .092	.005	− .062	.165^**^	− .028	.218^**^	.223^**^	.160^**^	.224^**^	.026	− .033	1				
15	.058	.035	.015	.109	.002	.035	− .034	.081	.089	.085	− .067	.151^**^	.127^*^	.191^**^	1			
16	− .030	− .042	− .054	− .026	− .144^*^	.232^**^	− .042	.336^**^	.259^**^	.435^**^	.123^*^	.105	.225^**^	.067	.174^**^	1		
17	.047	.100	.016	.077	− .090	− .026	.153^**^	.112	.055	.109	− .065	.164^**^	.158^**^	.134^*^	.166^**^	.220^**^	1	
18	− .081	− .152^**^	− .148^*^	− .018	− .244^**^	.193^**^	.065	.231^**^	.136^*^	.289^**^	.120^*^	.055	.125^*^	.142^*^	− .092	.225^**^	.147^*^	1

**Correlation is significant at the 0.01 level (2-tailed)

*Correlation is significant at the 0.05 level (2-tailed)

Structural indicators of the KMO (0.69) and Bartlett’s test of Sphericity (*P* < 0.001, *df* = 153, X^2^ = 618.35) verified interpretability of the EFA output.

Applying PCA and direct oblimin rotation in the performed EFA and based on the calculated eigenvalues (eigenvalue greater than 1 rule was assumed as the default retention criterion) and graphical representation of the resulted scree plot four latent factors were identified (Table 3) which explained 49.32% of the total variance.

**Table 3:** Factor loadings for the four identified factors in the validation study of parents’ cognitive perception inventory of disaster effects on children’s well-being (PCP-DCWB)

Items	Factor 1	Factor 2	Factor 3	Factor 4
	Loadings			
I feel that my child has become more fearful after the quake	0.76			
I feel that my child is always anxious	0.70			
There have been some changes in behavior of my children after the quake	0.69			
My child is become more dependent on us to do daily tasks such as bathing or cleaning	0.56			
After the quake, I feel that my child has become more aggressive and nervous	0.53			
My child likes to sleep beside us	0.48			
		Loadings		
I am dedicated to my responsibilities towards my family members		− 0.77		
At the moment, I am experiencing a purposeful life		− 0.74		
I have strong and close relationships with my family members		− 0.73		
Despite being exposed to the quake, I feel that I have an adequate control of my living conditions		− 0.68		
I am hopeful about the future		− 0.60		
			Loadings	
I become unhappy and sad when I think of that incident			− 0.64	
My child speaks frequently about the quake and its effects			− 0.57	
I can easily tell my feelings as well as circumstances, which I had after the quake, to other people			0.51	
				Loadings
My child suffers from nightmares after the quake				0.67
The educational attainment of my child has declined				0.60
I feel that my child likes to be alone				0.59
After the quake, my child has accustomed to chewing nails				0.52

Cronbach’s alpha and split-half reliability coefficients for the whole scale were 0.71 and 0.52 respectively showing a good internal consistency for the instrument. The estimated reliability measures for the identified four subscales (Table 4) also revealed good internal consistency of the items in factor 1 (perception about child mental health) and 2 (passion to help family members) but not in factors 3 and 4.

**Table 4:** Internal consistency (Cronbach’s alpha and split half) measures and cumulative variance explained for the identified subscales in the validation study of parents’ cognitive perception inventory of disaster effects on children’s well-being (PCP-DCWB)

Factors	Cronbach’s alpha	Split half	Cumulative variance explained
1	0.72	0.63	19.37
2	0.76	0.71	34.04
3	0.39	0.35	42.22
4	0.46	0.44	49.31

To label the identified factors in this study the authors were checked the loading patterns and pinpointed the items with greater loading values. At the next stage commonality of the items’ contents were determined and based on this procedure a label was associated with each factor considering the overall meaning of the factors and in accord with the theoretical and conceptual intent of the included items [33, 34].

Corrected item-to-total correlation indices (Table 5) within the four subscales of the PCP-DCWB i.e., “*perception about child overall well-being”*, “*coping with trauma’s long term effects”*, “*children or parents continuing memory of past disaster”* and “*perception about behavioral and educational problems”* were in the vicinity of the acceptable range (> 0.4) [28].

**Table 5:** Corrected item-to-total correlation indices within the four identified subscales in the validation study of parents’ cognitive perception inventory of disaster effects on children’s well-being (PCP-DCWB)

	Items	Item-to- total correlation indices
Factor 1: Perception about child mental health	I feel that my child has become more fearful after the quake	0.75
	I feel that my child is always anxious	0.75
	There have been some changes in behavior of my children after the quake	0.67
	My child is become more dependent on us to do daily tasks such as bathing or cleaning	0.51
	After the quake, I feel that my child has become more aggressive and nervous	0.62
	My child likes to sleep beside us	0.55
Factor 2: Passion to help family members	I am dedicated to my responsibilities towards my family members	0.75
	At the moment, I am experiencing a purposeful life	0.79
	I have strong and close relationships with my family members	0.68
Factor 3: Coping with trauma’s long-term effects	Despite being exposed to the quake, I feel that I have an adequate control of my living conditions	0.7
	I am hopeful about the future	0.64
	I become unhappy and sad when I think of that incident	0.73
	My child speaks frequently about the quake and its effects	0.66
	I can easily tell my feelings as well as circumstances, which I had after the quake, to other people	0.59
Factor 4: Perception about behavioral and conduct disorders	My child suffers from nightmares after the quake	0.65
	The educational attainment of my child has declined	0.64
	I feel that my child likes to be alone	0.6
	After the quake, my child has accustomed to nail-biting	0.56

## Discussion

This study aimed to develop a valid and reliable instrument for assessment of parents’ perception about disasters’ effects on children’s mental health. The EFA outputs yielded psychometric data to support a four-factor model to quantify parents’ cognition on well-being of children associated with experience of a devastating disaster. This could be a leap forward in better management of disasters’ health impacts and especially for maintaining the survived children’s well-being [24].

The reliability and the theoretical construct validity of the designed instrument were assessed, and the analyses output indicated that the tool is able to detect variations in the parents’ level of apprehension about psychological impacts of disasters on survived children’s well-being.

The constructed instrument (PCP-DCWB) with 18 items that dispersed in 4 latent factors (that explained about 49% of the total variance) can be considered for use as a single tool to measure parents’ perceptions about psychological impacts of disasters on their children or in conjunction with other instruments that measure specifically types or severities of post trauma psychological problems in children. The theoretical construct validity was verified and PCA separated the items into 4 themes. The content validity of the scale was also deemed to be satisfactory since the scale’s items reflect the dimensions that are described in the literature [6, 18, 20, 23, 24] as being main aspects of awareness parents must have about potentially negative psychological consequences of a disaster on children.

The overall estimates of content and construct validity and internal consistency measure of reliability for PCP-DCWB revealed almost satisfactory psychometric properties [35, 37, 38] however, the estimated reliability measures for two of the identified subscales (3 and 4) were below the range of expected values [37]. We know that alpha coefficient is sensitive to the number of items in a scale [37, 38] and it is likely that small number of items in the factors 3 and 4 (3 in the factor number 3 and 4 in the factor number 4) might resulted to small estimated alpha coefficient for these two latent factors. The observed low reliability measure for these two subscales therefore; might be explained by the small number of the included items that violate the tau equivalent model as the underlying fundament to estimate Cronbach’s alpha [39]. Additionally, when a small number of items with conceptual breadth are being categorized in a construct a lower alpha coefficient value can be expected [37]. Since establishing construct validity is an ongoing process and this study mainly focused on the theoretical dimensions of construct validity, further investigation and cross-validation is suggested in future to verify stability of these initial findings in other population samples.

Future studies should expand and assess applicability of the scale in different socio-cultural populations since parents’ cognition of disasters’ effects on psychological well-being of survived children in long term, can be affected differently by certain baseline socio-cultural attributes with direct impact on effectiveness of the interventions that target parents awareness in pre and post disaster preparation phases [33, 34, 40–49].

Children are more prone to the impacts of disasters than their parents and generally are neglected amid post disaster climate of agitation, disruption and discomforts [2, 3]. This scale development is an attempt to facilitate the understanding of parents’ perceptions about their children’s psychological well-being after a disaster and evokes importance of a thorough attention to the short and long term psychological impacts of disasters on survived children [28, 29].

The study findings must be interpreted by caution due to some limitations. The generalizability of the study findings might be limited by underrepresentation of those parents who were unwilling to participate in the study at the extremes of the socioeconomic status. Moreover, no data were collected on non-responders, which would have provided additional insights into the validity of the results and probability of non-response bias.

The recruited parents through their enrolled school children in two primary schools might not be representative of the all parents who survived the earthquake. The scale’s items were inherently structured to be self-completed and lack of formal cognitive interviews to assess the potential sources of response bias was another limitation of the study especially when considering the respondents’ level of education.

Several ethnic groups are living in Iran (Persians, Azeris, Kurds, Arabs, Baluchis, and Lurs) and Persian language is formal first language of the country. The study results were based on parents that were able to read and understand Persian and no data were collected relating to the participants’ mother language or ethnicity. Therefore, whether and how the respondents’ mother language or ethnicity may affect the results remains unknown [50, 51]. As this study was implemented as a preliminary psychometric testing of the PCP-DCWB no test–retest reliability was examined which is considered as an indicator of replicable and stable results.

There may also be differences in the type and severity of physical injuries, required care or management of the injured children that drew the parents’ special attention with impacts on the study outcomes.

The study participants were recruited about 3 years after the earthquake therefore; probability of recall bias due to the time lag should not be dismissed completely.

Some of the items related to psychological status of the parents were allocated to factors that have items which are directly related to the survived children’s well-being. Several reasons such as providing contradictory or inconsistent responses to the scale’s questions due to content or face validity issues, the applied data analysis method (use of principal component analysis (PCA) versus principal axis factoring as the extraction method) might have caused the inconsistency. Dropping an item form a list of extracted items that have selected based on an inclusive literature review was the last choice of option in this study. The developed scale’s application in other population samples with bigger data set is recommended to verify factor structure of the developed instrument and before deleting any item from the list of retrieved items.

Main advantage of the constructed scale is its items development based on an extensive literature search, ease of its application due to self-completion nature of the questions, relatively acceptable reliability and validity and limited time and energy burden on respondents that are considered important in developing instrument for health assessment in community-based surveys [30].

## Conclusion

Main focus in development of this instrument was on the most frequent and major signs of disordered well-being among survived children. This instrument was developed for use as a proxy measure for screening parents’ cognition about their disaster-impacted children’s overall psychological well-being. Disease specific instruments are recommended for application to detect those mental disorders that are common in post-disaster circumstances among survived children. The study findings suggested that the PCP-DCWB and its subscales retain almost acceptable internal consistency and construct validity. Outputs of factor analysis are generally sample specific and different results might be achieved in different studies. Therefore; further cross-cultural validation studies are warranted to assess the scale adaptability in other populations (disaster-affected and non-disaster affected) regionally and internationally. These initial findings however, suggest that the developed instrument (PCP-DCWB) might contributes to research methodology armamentarium in assessing parents’ cognitions about health consequences of disasters on child survivors and also as a proxy reporting tool of health interventions’ effectiveness over time.

This tool could also be applicable in recognizing and stratifying those families who may potentially be at a greater risk of emotional, behavioral or mental problems as the results of experiencing a traumatic event. Mental health professionals with being at the center of the care provision continuum to traumatized children, young people and their families therefore, could develop a trauma-informed and meaningful therapeutic relationship for empowering families based on genuine empathy and acceptance.

The PCP-DCWB may also be used to shed light on level of pre-disaster preparedness in local, national and international scales therefore; on the nature of intervention that are required to boost disaster preparedness in communities. Devastating natural and man-made disasters are occurring evenly or on a daily basis around the globe (wars, floods, tsunamis, thunderstorms etc.) and children are one of the major vulnerable age groups in the impacted regions [2, 3, 14, 15]. Thus, any attempt to maintain their psychological well-being after a disaster will pose considerable benefit in reconstruction and building of resilience among families in the affected areas.

## Data Availability

The datasets used and/or analysed during the current study are available from the corresponding author on reasonable request.

## Declarations

### Accordance statement

All methods were carried out in accordance with relevant guidelines and regulations.

## References

[1] Cullins LM, Mian AI (2015). Global child and adolescent mental health: a culturally informed focus. Child Adolesc Psychiatr Clin.

[2] Tang B, Liu X, Liu Y, Xue C, Zhang L (2014). A meta-analysis of risk factors for depression in adults and children after natural disasters. BMC Public Health.

[3] Piyasil V, Ketumarn P, Prubrukarn R, Pacharakaew S, Dumrongphol H, Rungsri S (2008). Psychiatric disorders in children at one year after the tsunami disaster in Thailand. J Med Assoc Thai.

[4] Commers MJ, Morival M, Devries MW (2012). Toward best-practice post-disaster mental health promotion for children: Sri Lanka. Health Promot Int.

[5] Kalantari M, Vostanis P (2010). Behavioural and emotional problems in Iranian children four years after parental death in an earthquake. Int J Soc Psychiatry.

[6] Cobham VE, McDermott B, Haslam D, Sanders MR (2016). The role of parents, parenting and the family environment in children’s post-disaster mental health. Curr Psychiatry Rep.

[7] Forresi B, Soncini F, Bottosso E, Di Pietro E, Scarpini G, Scaini S, Aggazzotti G, Caffo E, Righi E (2020). Post-traumatic stress disorder, emotional and behavioral difficulties in children and adolescents 2 years after the 2012 earthquake in Italy: an epidemiological cross-sectional study. Eur Child Adolesc Psychiatry.

[8] Orengo-Aguayo R, Stewart RW, de Arellano MA, Suárez-Kindy JL, Young J (2019). Disaster exposure and mental health among Puertorican youths after Hurricane Maria. JAMA Netw Open.

[9] Dhital R, Shibanuma A, Miyaguchi M, Kiriya J, Jimba M (2019). Effect of psycho-social support by teachers on improving mental health and hope of adolescents in an earthquake-affected district in Nepal: a cluster randomized controlled trial. PLoS ONE.

[10] Tang W, Zhao J, Lu Y, Zha Y, Liu H, Sun Y, Zhang J, Yang Y, Xu J (2018). Suicidality, posttraumatic stress, and depressive reactions after earthquake and maltreatment: a cross-sectional survey of a random sample of 6132 Chinese children and adolescents. J Affect Disord.

[11] Cheng J, Liang Y, Fu L, Liu Z (2018). Posttraumatic stress and depressive symptoms in children after the Wenchuan earthquake. Eur J Psychotraumatol.

[12] Hlodversdottir H, Thorsteinsdottir H, Thordardottir EB, Njardvik U, Petursdottir G, Hauksdottir A (2018). Long-term health of children following the Eyjafjallajökull volcanic eruption: a prospective cohort study. Eur J Psychotraumatol.

[13] Tang W, Zhao J, Lu Y, Yan T, Wang L, Zhang J, Xu J (2017). Mental health problems among children and adolescents experiencing two major earthquakes in remote mountainous regions: a longitudinal study. Comp Psychiatry.

[14] Arshad M, Mughal MK, Giallo R, Kingston D (2020). Predictors of child resilience in a community-based cohort facing flood as natural disaster. BMC Psychiatry.

[15] Al-Yagon M, Garbi L, Rich Y (2022). Children's resilience to ongoing border attacks: the role of father, mother, and child resources. Child Psychiatry Hum Dev.

[16] Angelkovski R (2016). Resilience in children: educational significance. J Student Engagem Educ Matters.

[17] Samuelson KW, Wilson CK, Padrón E, Lee S, Gavron L (2017). Maternal PTSD and children’s adjustment: parenting stress and emotional availability as proposed mediators. J Clin Psychol.

[18] Røkholt EG, Schultz J-H, Langballe Å (2016). Negotiating a new day: parents’ contributions to supporting students’ school functioning after exposure to trauma. Psychol Res Behav Manag.

[19] Lucio R, Nelson TL (2016). Effective practices in the treatment of trauma in children and adolescents: from guidelines to organizational practices. J Evid Inform Soc Work.

[20] Salloum A, Swaidan VR, Torres AC, Murphy TK, Storch EA (2016). Parents’ perception of stepped care and standard care trauma-focused cognitive behavioral therapy for young children. J Child Fam Stud.

[21] Nabors L, Baker-Phibbs C, Burbage M (2016). Predictors of child functioning and problem behaviors for children diagnosed with posttraumatic stress disorder and externalizing problems. J Prev Interv Commun.

[22] Gurwitch RH, Messer EP, Masse J, Olafson E, Boat BW, Putnam FW (2016). Child-adult relationship enhancement (CARE): an evidence-informed program for children with a history of trauma and other behavioral challenges. Child Abuse Negl.

[23] Sullivan KM, Murray KJ, Ake GS (2016). Trauma-informed care for children in the child welfare system: an initial evaluation of a trauma-informed parenting workshop. Child Maltreat.

[24] Abera M, Robbins JM, Tesfaye M (2015). Parents’ perception of child and adolescent mental health problems and their choice of treatment option in southwest Ethiopia. Child Adolesc Psychiatry Ment Health.

[25] Ghomian Z, Yousefian SH (2017). Natural disasters in the middle-east and North Africa with a focus on Iran: 1900 to 2015 health in emergencies and disasters quarterly. Winter.

[26] United Nations Office for Coordination of Humanitarian Affairs (OCHA). Middle East and North Africa (ROMENA): Iran. https://www.unocha.org/middle-east-and-north-africa-romena/iran.

[27] Aditi Bhavnani B, Burtonboy R, Hamad CH, Barandiaran OL, Safaie A, Tewari S, et al. Natural disasters in the Middle East and North Africa: a regional overview (English). Global Facility for Disaster Reduction and Recovery (GFDRR) Washington, D.C.World Bank Group. http://documents.worldbank.org/curated/en/211811468106752534/Natural-disasters-in-the-Middle-East-and-North-Africa-a-regional-overview.

[28] Terwee CB, Bot SDM, de Boer MR, van der Windt DAWM, Knol DL, Dekker J (2007). Quality criteria were proposed for measurement properties of health status questionnaires. J Clin Epidemiol.

[29] Atazadeh N, Mahmoodi H, Shaghaghi A (2019). Posttrauma psychosocial effects in children: a systematic review of measurement scales. J Child Adolesc Psychiatr Nurs.

[30] De Vellis RF (2016). Scale development: theory and applications.

[31] Shaghaghi A (2012). We need more focus on pre-disaster preparedness: early lessons learned from recent earthquakes in northwest of Iran. Heal Promot Perspect.

[32] Pfefferbaum B, North CS (2008). Children and families in the context of disasters: implications for preparedness and response. Fam Psychol Bull Div Fam Psychol.

[33] Pfefferbaum B, North CS (2013). Assessing children’s disaster reactions and mental health needs: screening and clinical evaluation. Can J Psychiatry.

[34] Pfefferbaum B, Varma V, Nitiéma P, Newman E (2014). Universal preventive interventions for children in the context of disasters and terrorism. Child Adolesc Psychiatr Clin.

[35] Polit DF, Beck CT, Owen SV (2007). Is the CVI an acceptable indicator of content validity? Appraisal and recommendations. Res Nurs Health.

[36] Anthoine E, Moret L, Regnault A, Sébille V, Hardouin J-B (2014). Sample size used to validate a scale: a review of publications on newly-developed patient reported outcomes measures. Health Qual Life Outcomes.

[37] Helms JE, Henze KT, Sass TL, Mifsud VA (2006). Treating Cronbach’s alpha reliability coefficients as data in counseling research. Couns Psychol.

[38] Tavakol M, Dennick R (2011). Making sense of Cronbach’s alpha. Int J Med Educ.

[39] Graham JM (2006). Congeneric and (essentially) tau-equivalent estimates of score reliability: what they are and how to use them. Educ Psychol Meas.

[40] Felix E, Hernández LA, Bravo M, Ramirez R, Cabiya J, Canino G (2011). Natural disaster and risk of psychiatric disorders in Puerto Rican children. J Abnorm Child Psychol.

[41] Felix E, You S, Vernberg E, Canino G (2013). Family influences on the long term post-disaster recovery of Puerto Rican youth. J Abnorm Child Psychol.

[42] Newman E, Pfefferbaum B, Kirlic N, Tett R, Nelson S, Liles B (2014). Meta-analytic review of psychological interventions for children survivors of natural and man-made disasters. Curr Psychiatry Rep.

[43] Garcia DM, Sheehan MC (2016). Extreme weather-driven disasters and children’s health. Int J Heal Serv.

[44] Bartenfeld MT, Peacock G, Griese SE (2014). Public health emergency planning for children in chemical, biological, radiological, and nuclear (CBRN) disasters. Biosecur Bioterrorism Biodefense Strateg Pract Sci.

[45] Kumar M, Fonagy P (2013). Differential effects of exposure to social violence and natural disaster on children’s mental health. J Trauma Stress.

[46] Kuwabara H, Araki T, Yamasaki S, Ando S, Kano Y, Kasai K (2015). Regional differences in post-traumatic stress symptoms among children after the 2011 tsunami in Higashi-Matsushima. Japan Brain Dev.

[47] Zhang Y, Zhang J, Zhu S, Du C, Zhang W (2015). Prevalence and predictors of somatic symptoms among child and adolescents with probable posttraumatic stress disorder: a cross-sectional study conducted in 21 primary and secondary schools after an earthquake. PLoS ONE.

[48] Latif F, Yeatermeyer J, Horne ZD, Beriwal S (2015). Psychological impact of nuclear disasters in children and adolescents. Child Adolesc Psychiatr Clin.

[49] Hayashi K, Tomita N (2012). Lessons learned from the Great East Japan Earthquake: impact on child and adolescent health. Asia Pacific J Public Heal.

[50] Mackay A, Ashworth M, White P (2017). The role of spoken language in cardiovascular health inequalities: a cross-sectional study of people with non-English language preference. BJGP Open.

[51] Handtke O, Schilgen B, Mösko M (2019). Culturally competent healthcare—a scoping review of strategies implemented in healthcare organizations and a model of culturally competent healthcare provision. PLoS ONE.

